# Transposable Elements: From DNA Parasites to Architects of Metazoan Evolution

**DOI:** 10.3390/genes3030409

**Published:** 2012-07-12

**Authors:** Oliver Piskurek, Daniel J. Jackson

**Affiliations:** Courant Research Centre Geobiology, Georg-August-University of Göttingen, Goldschmidtstr. 3, Göttingen 37077, Germany; E-Mail: opiskurek@web.de

**Keywords:** transposable element, junk DNA, molecular parasite, molecular domestication, functionalisation, exonisation, exaptation, SINE, LINE

## Abstract

One of the most unexpected insights that followed from the completion of the human genome a decade ago was that more than half of our DNA is derived from transposable elements (TEs). Due to advances in high throughput sequencing technologies it is now clear that TEs comprise the largest molecular class within most metazoan genomes. TEs, once categorised as "junk DNA", are now known to influence genomic structure and function by increasing the coding and non-coding genetic repertoire of the host. In this way TEs are key elements that stimulate the evolution of metazoan genomes. This review highlights several lines of TE research including the horizontal transfer of TEs through host-parasite interactions, the vertical maintenance of TEs over long periods of evolutionary time, and the direct role that TEs have played in generating morphological novelty.

## 1. Classification and Diversity of TEs

During her career Barbara McClintock discovered and described transposable elements (TEs), a class of mobile genetic elements often abundantly distributed throughout the genomes of eukaryotic organisms [1,2,3,4; reviewed in 5]. At the time, her findings were in line with the popular theory of selfish DNA in which TEs could be perceived as “genomic hitchhikers” or molecular parasites which play no significant role in genome evolution, and provide no adaptive advantage to the host [6,7]. Ahead of her time, Barbara McClintock nonetheless suggested that TEs can indeed influence the evolution of the genome. The ways in which this has since been shown to be true are amazing. These insights have been in part due to the rise of the field of evolutionary-developmental biology (evo-devo), rapid advances in DNA sequencing technologies and the concomitant rise in the field of comparative genomics. The modern day view of TEs is that they have the potential to act as agents of evolution by increasing, rearranging and diversifying the genetic repertoire of their hosts [8,9,10,11,12,13,14,15,16,17].

With significant advances in high throughput sequencing technologies has come the democratization of genome sequencing. Following the completion of the first metazoan genome in 1998 using 'first generation' technologies [18], draft genomes of "non-model" organisms are released with increasing annual frequency. With this flood of sequence data has come the need to develop bioinformatic tools designed to detect and characterise TEs [19,20,21]. TEs can be broadly divided into two classes based upon their mechanism of mobilisation or transposition (however the reader should be aware that newly described classes of TEs challenge this simplistic categorisation): class I elements (retroposons) mobilise via an RNA intermediate analogous to a “copy-and-paste” mechanism where the “copy” (RNA) is biochemically distinct from the original (DNA); class II elements (DNA transposons) mobilise via a DNA-mediated mode of transposition originally known as “cut-and-paste” mechanism. TEs of both classes can be classified as autonomous or non-autonomous, based on whether or not they encode the proteins necessary for their own retrotransposition/transposition. Four types of eukaryotic class I TEs can be distinguished [22,23]: Long terminal repeat elements (LTRs); non-LTR elements such as long interspersed elements (LINEs) and non-autonomous short interspersed elements (SINEs); and two types of TE with unusual structures, namely DIRS (based on DIRS-1, characterized in *Dictyostelium*) and Penelope-like elements (PLEs). TEs of class II can be divided into three major types: type 1 elements have two terminal inverted repeats (TIRs) and are typical cut-and-paste DNA transposons which are fully excised with the help of enzyme transposase; type 2 elements are rolling-circle DNA transposons also known as Helitrons [14,24]; and type 3 elements which are self-synthesizing DNA transposons, also known as Polintons or Mavericks [25].

TEs can often be recognised as genomic fossils that were once autonomously replicating elements which at some point in time experienced a deletion, inversion, or other mutation that rendered them inactive. Alternatively, a ‘fossilised non-autonomous TE’ can remain active as long as the enzymatic machinery required for transposition is provided by an autonomous partner. A good example of this is the LINE-SINE system. The 3′ tail sequence of a SINE is identical to that of its partner LINE and is recognized by the reverse transcriptase (RT) of that LINE [26,27,28]. Thus, when SINEs replicate via retrotransposition they depend on the existing retrotranspositional machinery of their active LINE partners. The largest TE class within most metazoan genomes consists of LINEs and SINEs [29,30]. For example, the LINE1-*Alu*-SINE system comprises nearly 30% of the human genome [31,32]. In marsupials LINEs and their related mobilized SINEs make up nearly 40% of the genome [33,34], while LINEs/SINEs in the lizard genome (including the Bov-B LINE-Sauria SINEs system) represent about 17% of the total DNA [35,36,37]. Based on these observations Hua-Van *et al.* [38] discuss the concept of a struggle for survival between TE families, similar to that which occurs between species sharing the same ecological niche.

Current views of metazoan TE diversity and distribution are likely to be biased and under-representative. This is because genomes of direct relevance to human medicine or evolution, or of unusually small size are preferentially sequenced. Furthermore, comprehensive and accurate identification and annotation of the TE complement in any metazoan genome requires: (i) a high quality genome assembly and; (ii) bioinformatic tools that can recognise both conserved (typically homology based methods) and novel (*i.e.*, *de novo* detection methods) TEs. Both of these requirements are non-trivial. Continuing technical advances in sequencing and *in silico* assembly methods could be expected to eventually eliminate the first problem. Detecting truly novel, lineage specific TEs using *de novo* methods is an inherently challenging bioinformatic exercise (relative to homology based methods). However, with the sequencing of more phylogenetically representative taxa from the metazoan tree of life for evolutionary studies [39,40,41], and the corresponding development of tools to annotate these datasets [42,43,44], it can be expected that in the near future we will come to appreciate that the evolutionary histories and functions of TEs are as complex and diverse as the biological populations bearing them.

## 2. DNA Transposons—Horizontal Transfer Events Facilitate the Spread of TEs

Horizontal transfer (HT), the exchange of genetic material between two species that do not share an immediate ancestor-descendant relationship, of a TE from one genome to another can trigger many molecular events, which can in turn directly influence genome evolution. Furthermore, HT is an effective strategy that ensures the long-term survival of an active DNA transposon. This is because HT allows the element to evade extinction in the host which may be brought about by host repression of TE activity, or by extinction of the host lineage. The phenomenon of HT of genetic material between bacteria is well known. This mechanism can explain the abundance of insertion sequences (IS) in prokaryotic genomes [45]. However, HT events across domains of life, *i.e.*, from bacteria to eukaryotic organisms, are far less common. The genomes of bdelloid rotifers appear to have evolved for millions of years without sexual reproduction, and possess genes thought to have been acquired by HT from bacteria, fungi, and plants [46]. Concerning TEs, there is only one known HT event from a prokaryote to a eukaryote, namely the IS5-like integration from a bacterium into a bdelloid rotifer genome [47]. This HT event apparently took place recently as the TE has not increased in copy number within the bdelloid genome.

Classical examples of horizontally transferred TEs between metazoans include the P-elements in *Drosophila* [48], the Mariner transposons in insects [49], and the chromoviruses, the oldest and largest lineage of LTR elements, which were horizontally transferred into the genome of the ancestor of gnathostomes [50]. Although these and other class II TEs are well adapted to invade species via horizontal transmission [51], it has been suggested that all types of TEs may be subject to HT [52,53,54]. Schaack *et al.* [54] lists more than 100 cases of HTs in which class I TEs are involved, mostly LTR and non-LTR elements. A typical example for the HT of non-LTR elements is the Bov-B LINE which was initially discovered in ruminants but later shown to be ubiquitous in squamate genomes [55,56]. Given the patchy phylogenetic distribution of Bov-B LINEs among mammalian genomes it was inferred that these TEs were transferred horizontally from an ancestor of derived snakes to an ancestor of ruminants. A potential vector for this transfer was discovered when a Bov-B-derived Sauria SINE from the snake *Echis ocellatus* was identified in a poxvirus known to infect mammals [57].

Apart from viruses and bacteria, eukaryotic parasites can also facilitate the spread of TEs across diverged host species. Houck *et al.* [58] proposed that the mite *Proctolaelaps regalis* may serve as a vector for the HT of TEs between different *Drosophila* species. Furthermore, it was recently shown that the genome of *Rhodnius prolixus*, a triatomine bug which feeds on the blood of diverse tetrapods, harbours four DNA transposon families which are also present in the genomes of the bug's preferred hosts [54,59]. Finally, the hookworm *Ancylostoma caninum*, a parasite of dogs that is frequently detected in the human small intestine, harbours a mariner-like DNA transposon (bandit) that is phylogenetically related to the human Hsmar DNA transposon, suggesting that a HT of bandit may have taken place between hookworm parasites and mammalian hosts [60]. The complexity of TE evolution is highlighted in these cases where the TE and the host genome co-evolve in parallel with the co-evolutionary arms race of the parasite and host [61]. In order to fully understand how the genomic TE complement of a given eukaryotic organism evolved, it is therefore often necessary to understand the ecology of any host-parasite interactions that organism may have. Nonetheless, because TEs can become functionally relevant for the host genome (see Section 4 below), the exchange of such genetic material between species, regardless of how it is delivered, can have a striking impact on genome evolution.

## 3. Retroposons—TEs as Molecular Markers to Infer Phylogenetic Relationships

While horizontally transferred DNA transposons can provide information concerning the ecological interactions of host species, retroposons are usually vertically inherited and can therefore provide information concerning the phylogenetic relationships of species. Using SINE/LINE partners to infer phylogenetic relationships by parsimony is a powerful method when multiple retroposon insertions show the same phylogenetic pattern [62,63]. This method simply treats an insertion at a specific genomic location as a derived character state, while the lack of an insertion at an orthologous locus is regarded as the ancestral state. SINEs and LINEs are suitable for phylogenetic studies for several reasons: they insert almost randomly into genomic DNA; most copies are non-autonomous; they exist in large copy numbers; and their transfer usually occurs vertically. For more than a decade, SINEs and LINEs have been successfully employed as molecular markers, particularly by the Okada group [64,65,66,67,68,69] and Schmitz and colleagues [34,70,71,72,73]. It is also known that retroposons can reveal rapid radiation (incomplete lineage sorting) events as shown recently for the origin of placental mammals [69,72]. In this case retroposons were not fixed in the ancestral population before the separation of lineages (incomplete lineage sorting), and therefore cannot be used as phylogenetic markers. Such situations can nonetheless provide insight into the evolutionary and geological history of the three placental lineages (Afrotheria, Xenarthra, and Boreotheria). These lineages divided nearly simultaneously in parallel with the division of continents that lead to isolated Africa, South America, and Laurasia. Such TE insertion polymorphisms are not informative for phylogenetic analyses, but they do provide an efficient tool for the identification of rapid speciation events [63,74]. For example, recent high-throughput sequencing and comparison of human genomes revealed extensive variation in LINE1 content [42], illustrating that such TEs can not only be used as phylogenetic markers but are a major source of individual genomic variation.

## 4. TEs Are a Source of Novel Genetic Material

TEs are often associated with genome expansions and increases in genomic diversity (e.g. 85% of the maize genome, 57% of the *Hydra* genome, and 45–69% of the human genome are composed of TEs) [31,75,76,77]. In many cases the insertion of a TE has either no impact (typical for insertions into non-functional DNA regions), or deleterious effects as a result of disrupting coding DNA or gene regulatory regions such as promoters. TE insertions can also cause sequence inversions, duplications or deletion events and are therefore a potential source of genetic diseases [78,79]. Deleterious TE activity can also be brought about by the ectopic recombination of non-homologous regions of a chromosome. Such rearrangements during meiosis can produce unviable gametes, and it has been recently shown in *Drosophila* that selection against such events is the major force driving TE population dynamics [80].

SINEs, LINEs and other transposed sequences can however positively influence the host genome in many ways. For example, Jordan *et al.* [81] analysed promoter regions in the human genome and found that almost 25% contain TE-derived sequences associated with transcriptional regulation, while Nekrutenko and Li [82] examined 13,799 human genes and found 533 genes associated with TE insertions, of which these were mostly SINEs (~40%) and LINEs (~27%). As a specific example of this, Lunyak *et al.* [83] showed that tissue-specific transcription of a SINE sequence in the murine growth hormone locus is required for the establishment of functional chromatin domains, which in turn permit gene activation. It is now clear that TE insertions into the untranslated regions of genes are frequently associated with alternative splicing events (exonizations), and the *de novo* generation of exons [84,85,86,87]. In addition to exonization, TE insertions are also known to deliver novel introns. Indeed TE mediated intron insertions are thought to be responsible for much of the wide-spread intron gains observed in mammalian genomes [88]. TEs that evolve into novel protein coding sequences by exonization might subsequently acquire a function in a process called exaptation [8,89]. Several criteria can be used to detect whether such domestication of a TE has taken place: (i) evidence of TE fixation in a population; (ii) the presence of an intact open reading frame and splice sites; (iii) the presence of orthologous TE sequences in several species. Comparative analyses of mammalian genomes have revealed that only 1.5–2.0% of the human genome constitute protein coding genes, while up to 5% of the genome consists of conserved non-coding elements (CNEs) [90]. Two recent studies characterized certain SINEs which make up this population of CNEs: Bejerano *et al.* [12] identified the living fossil LF-SINE in the coelacanth, while Nishihara *et al.* [66] found homologous AmnSINE1 members in amniotes. Some of the exapted SINE copies, which accumulated mutations over evolutionary time, form ultra-conserved enhancers, such as the LF-SINE locus 0.5 Mb upstream of the neuro-developmental gene *ISL1* [12]. Recently, the genome of the marsupial *Monodelphis domestica* revealed that at least 16% of eutherian-specific CNEs are derived from TEs [33]. It has been suggested that phylogenetically conserved SINEs with conserved domains, such as the CORE-SINEs found in bilaterians [91], V-SINEs in vertebrates [92], DeuSINEs in deuterostomes [66], and CephSINEs in cephalopods [93], can act as functional modules that enhance gene expression. Some recent studies have indeed experimentally demonstrated this to be true [15,16,94,95]. Interestingly, these examples of TE exaptation are often associated with morphological innovations. Santangelo *et al.* [94] identified an ancient exaptation of a CORE-SINE that has remained under purifying selection in all mammalian orders for the last 170 million years. This CORE-SINE locus, as well as the recently identified exapted LTR locus in placental mammals [95], function as *cis*-regulatory elements that regulate hormone activity of the proopiomelanocortin gene. Sasaki *et al.* [15] also used a transgenic mouse system to show that the AmnSINE1 loci AS071 and AS021 were exapted about 300 million years ago in a common ancestor of reptiles, birds, and mammals to a role in forebrain development. It has been proposed that this biological innovation allowed these lineages to more readily adapt to the low oxygen concentrations (~ 10%) that predominated after the Permian-Triassic mass extinction 250 million years ago [96]. Another striking example of the ability of TEs to influence the evolution of morphological novelties is that of the MER20 locus (hAT-Charlie family DNA transposon) which possibly contributed to the origin of a novel gene regulatory network dedicated to pregnancy in placental mammals [97]. These examples clearly illustrate that TEs are able to directly influence genomic and morphological evolution [13,98,99,100].

## 5. TE Research in “Under-Represented Clades from the Metazoan Tree of Life”

As the number of publicly available non-model genomes increases, so too does the diversity of bioinformatic tools designed to analyse them. Molecular biologists interested in identifying TEs in a novel genome *can choose from a large* number of bioinformatic methods designed to screen for these elements in large datasets [19,43,44,101,102,103,104,105,106,107,108]. Because the fundamental approaches employed by these algorithms can vary dramatically, carrying out a comparison of the results they can generate appears to be a suitable way of acquiring reliable results [19,44]. For example, a combined screening approach with PILER [102] and RepeatScout [105] was used recently to identify TEs in the first reptilian genome to be sequenced, the green anole lizard *Anolis carolinensis* [37]. TEs total 30% of this reptilian genome, and display a much wider variety of TE families than was previously recognized in the genomes of birds and mammals [109] with LINEs and SINEs being the most abundant TEs [37,110]. While many of these TEs are still active in the lizard (which probably reflects the ancestral condition of the amniote ancestor), 96 of these were identified as having been exapted in the human genome. For example, Alföldi *et al.* [37] identified a protein-coding exon that is highly conserved across 29 mammals, and was exapted from a LINE2 sequence that is now part of the MIER1 (mesoderm induction early response 1) protein in mammals. TEs are also known to be involved in the evolution of venom toxin genes in reptiles [111]. For instance, introns of the PIII-SVMP gene in the highly poisonous snake *Echis ocellatus* contain a number of different LINEs, Sauria SINEs, and a hAT transposon which may have contributed to the functional recruitment and duplication of this gene in the venom gland [112].

Further examples of the influence of TEs on the evolution of non-model genomes can be found in the loci of four *Hox* genes (the products of which control many highly conserved aspects of embryonic development across the Metazoa) in the green anole lizard. These loci are known to have massively accumulated TEs [113], mostly PLEs [114] and Sauria SINEs [35]. This is especially interesting because *Hox* genes are thought to lack TE sequences in other vertebrates [115]. Considering this unique situation in vertebrates, and because TEs are also present in other developmental gene regions in squamate reptiles (lizards and snakes), a correlation between TE activity and the morphological diversity of squamate species has been postulated [110,113]. In a related example, the ParaHox clusterin the genome of the cephalochordate *Branchiostoma floridae* is known to be a hotspot for TE insertion [116] illustrating that not all genomic regions are equally receptive to TE invasion.

Phylogenetically broad searches for TEs based on recent genome releases have identified highly conserved metazoan retroposons. For example, de la Chaux and Wagner [117] identified BEL/Pao retroposons in 53 metazoan genomes, including the sponge genome (*Amphimedon queenslandica*) [118], and concluded that these elements evolved during early metazoan evolution. In contrast, the genome of the phylogenetically enigmatic *Trichoplax* [119] was recently shown to be relatively devoid of conserved TEs [120]. In our own efforts to identify highly conserved and ancient TEs we characterized SINE sequences with a deeply conserved domain (the Nin-domain) in the genomes of cnidarians, molluscs, annelids, and arthropods. The Nin-domain can be traced back to the origins of the Eumetazoa > 600 million years ago, making this SINE domain the most phylogenetically widespread, vertically transferred SINE sequence currently known [121]. Going further back in time, DIRS1-like and PLE retroposons are thought to have emerged during the radiation of the eukaryotes [122,123]. However, DIRS1-like TEs remain undetected in streptophytes and mammals, while PLEs have not been detected in mammals.

## 6. Conclusions

It is a testament to their diversity and pervasiveness that after 60 years of their discovery TEs remain scientifically topical. Even in genomic datasets that have long been available, novel TEs with high intra-genomic copy numbers and signatures of deep evolutionary conservation are still being identified. This is in part due to the relatively recent appreciation that TEs in fact have the capacity to directly influence functional genomic output. This paradigm shift away from the notion of TEs being parasitic “junk-DNA”, coupled with the growth of the fields of evo-devo, comparative genomics and concomitant technical advances in DNA sequencing technologies, has yielded great insights into the mechanisms of how complex genomes and morphological novelty can evolve. As whole genome datasets from ‘exotic’ metazoans continue to accumulate, we can expect the rate and breadth of TE discovery to accelerate and deepen. What is difficult to predict is the new ways in which TEs will be shown to interact with and influence their host genomes.
